# Ammonium ions improve the survival of glutamine-starved hybridoma cells

**DOI:** 10.1186/s13578-016-0092-8

**Published:** 2016-04-14

**Authors:** Abdelmuhsen Abusneina, Eric R. Gauthier

**Affiliations:** Biomolecular Sciences, Laurentian University, Sudbury, ON P3E 2C6 Canada; Department of Chemistry and Biochemistry, Laurentian University, Sudbury, ON P3E 2C6 Canada; Department of Biology, Laurentian University, Sudbury, ON P3E 2C6 Canada

**Keywords:** Ammonium, Apoptosis, Caspase, Glutamine, Glutaminolysis, Hybridoma

## Abstract

**Background:**

As a consequence of a reprogrammed metabolism, cancer cells are dependent on the amino acid l-glutamine for their survival, a phenomenon that currently forms the basis for the generation of new, cancer-specific therapies. In this paper, we report on the role which ammonium ions, a product of glutaminolysis, play on the survival of l-glutamine-deprived Sp2/0-Ag14 mouse hybridoma cells.

**Results:**

The supplementation of l-glutamine-starved Sp2/0-Ag14 cell cultures with either ammonium acetate or ammonium chloride resulted in a significant increase in viability. This effect did not depend on the ability of cells to synthesize l-glutamine, and was not affected by the co-supplementation with α-ketoglutarate. When we examined the effect of ammonium acetate and ammonium chloride on the induction of apoptosis by glutamine deprivation, we found that ammonium salts did not prevent caspase-3 activation or cytochrome c leakage, indicating that they did not act by modulating core apoptotic processes. However, both ammonium acetate and ammonium chloride caused a significant reduction in the number of l-glutamine-starved cells exhibiting apoptotic nuclear fragmentation and/or condensation.

**Conclusion:**

All together, our results show that ammonium ions promote the survival of l-glutamine-deprived Sp2/0-Ag14 cells and modulate late-apoptotic events. These findings highlight the complexity of the modulation of cell survival by l-glutamine, and suggest that targeting survival-signaling pathways modulated by ammonium ions should be examined as a potential anti-cancer strategy.

## Background

When compared to their quiescent counterparts, highly proliferating cells are characterized by a drastically different metabolic profile [1]. One of the most dramatic changes is the shift from the complete degradation of glucose through glycolysis and the Krebs cycle (to generate energy) to the use of glucose for the generation of biosynthetic precursors (through aerobic glycolysis, the so-called Warburg effect) [1, 2]. This is accompanied by an increase in the catabolism of the amino acid l-glutamine (Gln), which is used to replenish the Krebs cycle through anaplerosis, to obtain energy, and for the production of biosynthetic precursors and reducing co-factors [3]. These shifts in metabolism are driven by changes in the expression and activity of metabolic enzymes through a complex regulatory network involving (proto) oncogenes/tumor suppressors (e.g. HIF-1α, c-MYC and p53), protein kinases and phosphatases (e.g. AKT), sirtuins and microRNAs [4, 5]. As a consequence of metabolic reprogramming, highly proliferating cells (including cancer cells) become dependent on Gln for their growth and survival, a phenomenon called Gln addiction [6]. The latter forms the basis for the design of new therapeutics that specifically target cancer cells [6, 7].

The importance of Gln for cell survival has been recognized since the 1950s [8]. Gln has been shown to protect against cell stress, for example, through the up-regulation of heat shock proteins [9] or the inhibition of stress-related pathways [10, 11]. Moreover, Gln deprivation was shown to trigger apoptosis in several normal and transformed cell types including lymphocytes, neutrophils and intestinal epithelial cells [12]. How cells sense Gln and/or its metabolites and relay this information to the cell death/survival machinery is still not completely understood. In some cell lines, Gln regulates survival through mechanisms that do not require its catabolism. For instance, the decrease in intracellular Gln following its withdrawal has been shown to trigger an osmotic imbalance, the resulting reduction in cell volume leading to the ligand-independent activation of the extrinsic (i.e. death receptor-mediated) apoptotic pathway [13]. In other cases, the efflux of intracellular Gln is used to power the influx of leucine, which in turn activates the mTOR pathway, a central regulator of cell growth and survival [14–16].

In other situations, the promotion of cell survival by Gln requires prior processing of the amino acid through catabolic pathways. As a precursor for glutathione, Gln participates in protecting cells from oxidative stress and death [10]. Moreover, recent studies have revealed that α-ketoglutarate (αKG), generated through glutaminolysis, as well as other Krebs cycle intermediates promote the viability of Gln-deprived cells [17–19]. This pro-survival effect is, at least in part, the result of the synthesis of the amino acid asparagine [19] and the inhibition of cell death pathways by αKG [20].

On the other hand, it is generally recognized that another product of glutaminolysis, the ammonium ion (NH_4_^+^), is detrimental to cells. Its effects vary according to cell types, from functional disruption [21, 22] to growth inhibition [23, 24] and death [25–28]. However, ammonium ions have also been shown to prevent the induction of apoptosis [29, 30], raising the possibility that the perturbations in ammonium ion metabolism occurring under situations of Gln starvation could contribute to the initiation of death pathways.

In this study, we examined the role played by ammonium ions in the modulation of cell survival following Gln starvation. To this end, we used the mouse hybridoma Sp2/0-Ag14 (Sp2/0), a cell line that undergoes apoptosis through the intrinsic pathway within minutes of Gln deprivation [31]. Our data demonstrate that ammonium ions significantly increase the viability of Gln-starved Sp2/0 cells, cause a significant decrease in apoptotic nuclear condensation and fragmentation but have no detectable effect on the core apoptotic machinery. Therefore, ammonium ions, one of the two products of glutaminolysis, contribute to the modulation of cell survival by Gln.

## Methods

### Cell lines and reagents

Unless specified, all reagents were purchased from Sigma-Aldrich (Oakville, ON). The Sp2/0 mouse B cell hybridoma (ATCC CRL-1581) and the human Ramos B cell line (ATCC CRL-1596) were obtained directly from the American Type Culture Collection (Rockville, MD). All antibodies were from Cell Signaling Technology (Danvers, MA). l-Gln was prepared as a 200 mM stock solution in PBS (9.1 mM Na_2_HPO_4_, 1.7 mM NaH_2_PO_4_, 150 mM NaCl, pH 7.4) and adjusted to pH 7.4. Solutions of ammonium acetate (AA), ammonium chloride (AC), sodium acetate (SA) and sodium chloride (SC) were prepared as 5 M stocks in PBS and adjusted to pH 7.4.

### Cell culture

Sp2/0 cells were cultured in Iscove’s Modified Dulbecco’s medium (IMDM– Fisher Scientific, Ottawa, ON) supplemented with 1 % antibiotics (10 U/mL penicillin and 100 µg/mL streptomycin), 5 % Fetalclone I (Fisher Scientific) and 4 mM l-Gln. This medium will thereafter be referred to as complete IMDM. Ramos B cells were cultured in complete IMDM in which 10 % Fetal Bovine Serum (Fisher Scientific) replaced the Fetalclone I. The cultures were maintained at 37 °C in a humidified atmosphere of 5 % CO_2_ and 95 % air.

For the Gln-deprivation experiments, cells were washed twice in warm IMDM containing 1 % antibiotics and 5 % Fetalclone I but no Gln (referred to as Gln-free IMDM). The cells were then placed in Gln-free IMDM at a concentration of 4 × 10^5^ cells/mL and cultured for 3 h. Where indicated, cultures were supplemented with 5 mM of AA or AC at the start of the experiment. As controls, cultures were supplemented with PBS, 5 mM of SA or SC. Methionine sulfoximine (MSO, 200 mM stock dissolved in PBS) and dimethyl-α-ketoglutarate (diMe–α-KG, 100 mM stock dissolved in ethanol) were used at final concentrations of 10 and 2 mM, respectively. Controls in which Gln (4 mM) was added at the start of the culture were included in each experiment.

For the determination of cell viability using the trypan blue dye exclusion assay, the cells were processed for Gln deprivation as described above. After 3 h of Gln starvation, the cells were washed twice, resuspended in complete IMDM, and cultured for 24 h. Cell viability was then determined using the trypan blue dye exclusion assay.

For the clonogenic assays, the cells were processed for Gln deprivation for 3 h, after which time they were diluted in complete IMDM and seeded at a concentration of 0.5 cells/well in a 96-well plate. The cells were cultured for 7–10 days, and wells containing colonies of at least 50 cells were enumerated.

The analysis of cell viability using the Annexin V-FITC (AnnV) Apoptosis kit from Biovision Inc. (Milpitas, CA) was performed following the instructions from the manufacturer. FITC and propidium iodide (PI) fluorescence readings were taken using a BD FACSCanto II flow cytometer and the BD FACSDiva analysis software (BD Biosciences, Mississauga, ON), using the FL1 filter for FITC and the FL2 filter for PI. Cells which were negative for AnnV and PI staining were categorized as viable.

### Apoptosis assays

The activity of caspase-3 was measured using a colorimetric caspase-3 assay kit from Biovision Inc., following the instructions from the manufacturer. Absorbance readings were collected using a PowerWave-X microplate spectrophotometer (BioTek Instruments Inc., Winooski, VT).

For the analysis of apoptotic nuclear condensation and fragmentation, cells were processed for a 3 h Gln deprivation experiment, as described above. Thirty minutes before the end of the incubation period, Hoechst 33342 was added to the culture at a final concentration of 2 µg/mL. The cells were then washed twice with PBS and observed under fluorescence microscopy using a Zeiss Axiovert 200 M microscope (Carl Zeiss Canada Ltd, Toronto, ON).

The analysis of cytosolic cytochrome c release and DNA fragmentation was performed as described by Paquette et al. [31].

### Western blotting

Sp2/0 cells were processed for a 3 h Gln starvation experiment as described in the text, and washed twice in cold PBS. Whole cell extracts were prepared in RIPA cell lysis buffer (1 % IGEPAL, 0.5 % sodium deoxycholate, 0.1 % SDS, 0.2 mM sodium orthovanadate, 50 mM sodium fluoride, 0.1 mg/mL phenyl methyl sulfonyl fluoride and HALT protease inhibitor cocktail). Cytosolic extracts were prepared as described [31, 32]. For the analysis of PARP and Lamin A/C cleavage, extracts were prepared using a urea lysis buffer (62.5 mM Tris–HCl, pH 6.8, 6 M urea, 10 % glycerol, 2 % SDS, 0.00125 % bromophenol blue and 5 % 2-mercaptoethanol) [33]. Proteins were fractionated by SDS-PAGE and transferred onto an Immobilon-P membrane (EMD Millipore Corporation, Etobicoke, ON). Transfer efficiency was verified routinely by staining the membrane with Ponceau S. Western blot analysis was performed by diluting the primary antibodies 1/1000 in Tris-buffered saline Tween-20 (0.02 M Tris–HCl, 0.14 M NaCl, 0.1 % Tween 20, pH 7.6) buffer containing 5 % bovine serum albumin. Primary antibodies against the following proteins were used: caspase 3 (cat.# 9962), lamin A/C (cat.# 2032), β-actin (cat.# 4967), cytochrome c (cat.# 11940) and PARP (cat.# 9542). All the antibodies were polyclonal, with the exception for cytochrome c, which was a monoclonal antibody. The secondary antibody consisted in a polyclonal goat anti-rabbit IgG antibody coupled to horseradish peroxidase (cat.# 7074; diluted 1/5000 in Tris-buffered saline Tween-20 containing 5 % bovine serum albumin). Detection was performed by chemiluminescence using a Immobilon Western Reagent (EMD Millipore) and a Fluorchem 8000 Imaging system (Alpha Innotech, San Leandro, CA).

### Statistical analysis

Statistical analysis was performed using a two-tailed Student’s *t* test. Statistical significance was set at p < 0.05. Data are expressed as average ± standard deviation.

## Results

### Ammonium ions improve the viability of Gln-starved Sp2/0 cells

**Fig. 1:**
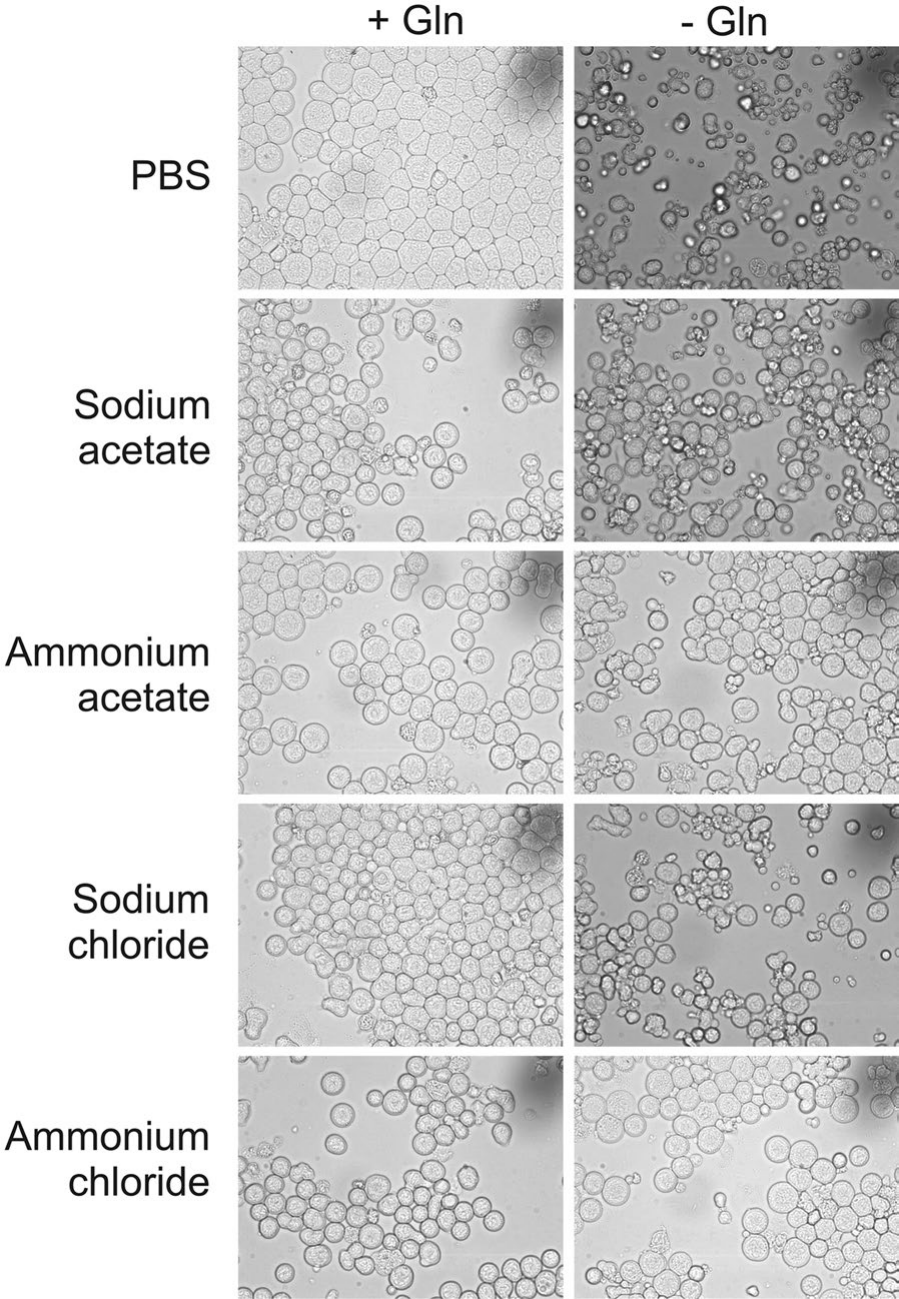
Effect of ammonium salts on the morphology of glutamine-starved Sp2/0-Ag14 cells. Cells were deprived of l-glutamine (−Gln) for 3 h in the presence of 5 mM ammonium salt/sodium salt or an equivalent volume of PBS. Cultures in which Gln was added at the start of the experiment (+Gln) were included as controls. magnification: ×400

We first examined whether ammonium ions influenced the viability of Sp2/0 cells subjected to Gln starvation. Compared to the Gln-fed control, Sp2/0 cells deprived of Gln and supplemented with PBS had a morphology characteristic of apoptotic cells, with a shrunken cytoplasm and the formation of apoptotic bodies (Fig. 1). Gln-starved cells supplemented with SA or SC also showed apoptotic morphology. However, the Gln-deprived Sp2/0 cultures in which cells were treated with AA or AC contained a greater proportion of cells with a normal morphology, suggesting that ammonium salts afforded some protection against the induction of cell death.

**Fig. 2:**
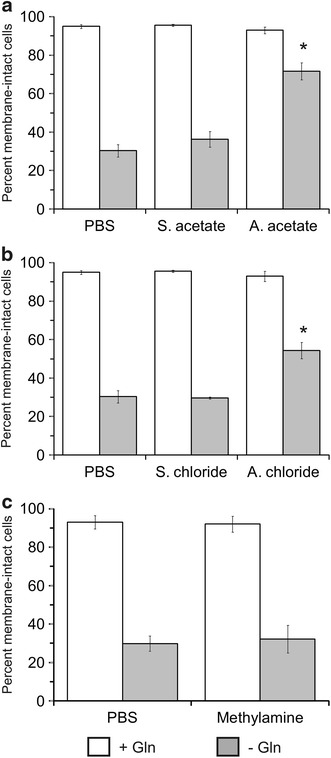
Ammonium salts improve the viability of glutamine-starved Sp2/0-Ag14 cells—trypan blue exclusion assay. Cells were deprived of l-glutamine for 3 h in the presence of 5 mM ammonium salt/sodium salt/methylamine chloride or an equivalent volume of PBS. Cell viability was determined by trypan blue exclusion. Cultures in which Gln was added at the start of the experiment were included as controls. *p < 0.05 vs corresponding sodium salt-supplemented control

These observations were validated in a trypan-blue dye exclusion assay. Sp2/0 cultures deprived of Gln for 3 h and supplemented with PBS showed 30 % cell viability (Fig. 2a, b), which is in accordance with our previous reports [31, 34]. Culture supplementation with the sodium salts yielded similar results. However, a significant improvement in cell viability was achieved when AA or AC were added to the cultures, with 72 % and 54 % viable cells, respectively. The pro-survival effect of ammonium ions was not observed when methylamine chloride was used (Fig. 2c). All the control cultures that were supplemented with Gln at the start of the experiment had cell viabilities above 90 %, indicating that the ammonium and sodium salts were not, on their own, toxic to the cells.

**Fig. 3:**
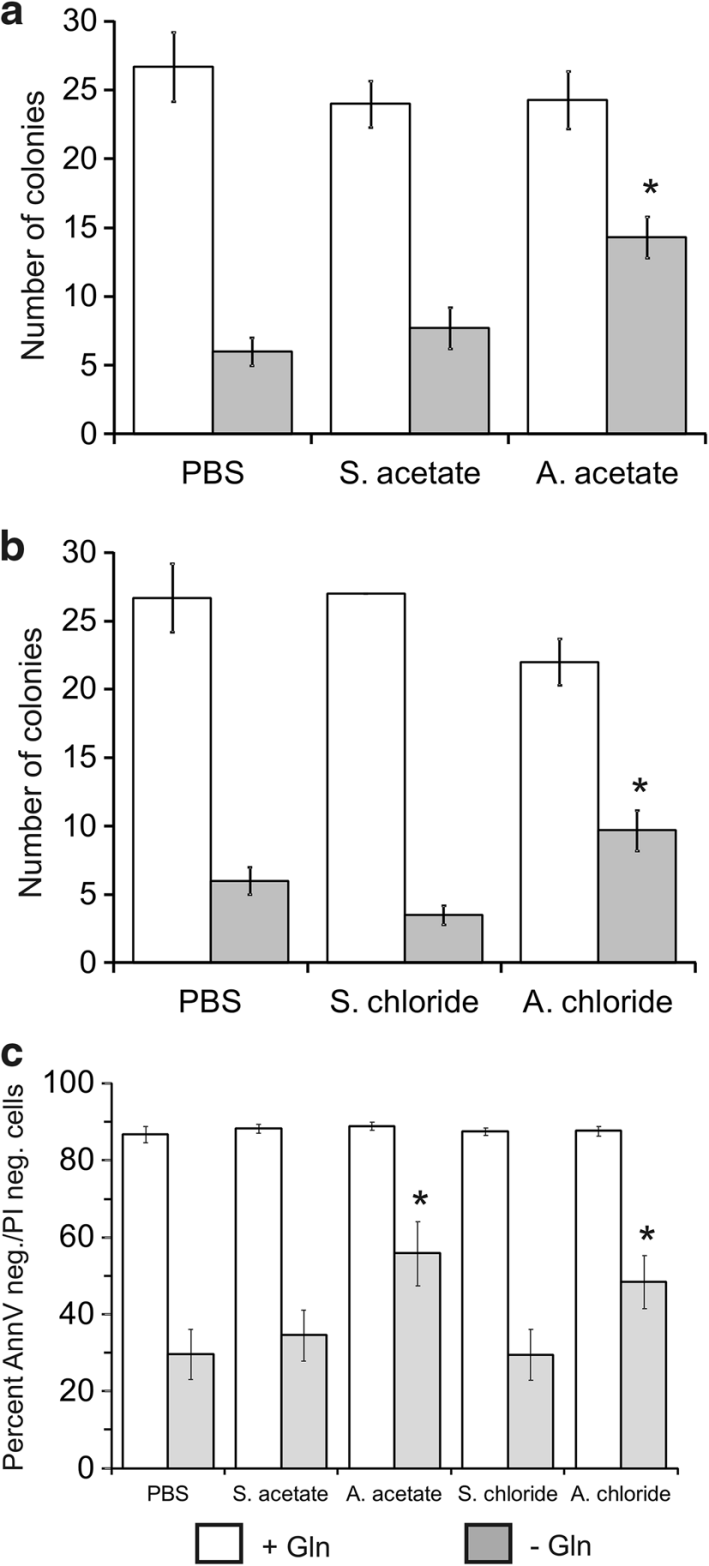
Ammonium salts improve the viability of glutamine-starved Sp2/0-Ag14 cells—clonogenic and flow cytometry assays. Cells were deprived of l-glutamine for 3 h in the presence of 5 mM ammonium salt/sodium salt or an equivalent volume of PBS. Panels **a** and **b**, clonogenic assay. Panel **c**, Annexin-V/propidium iodide assay. Cultures in which Gln was added at the start of the experiment were included as controls. *p < 0.05 vs corresponding sodium salt-supplemented control

These data were confirmed when we performed clonogenic assays (Fig. 3a, b). A significant increase in the number of Sp2/0 cell colonies over the PBS and sodium salt-supplemented controls was obtained when AA or AC were added to Gln-starved cultures. Finally, flow cytometry analysis using AnnV/PI staining revealed a significant increase in the number of viable cells (i.e. AnnV-negative, PI-negative) in cultures deprived of Gln and supplemented with ammonium salts (Fig. 3c). All together, these data show that ammonium salts promote the survival of Gln-starved Sp2/0 cells.

### Ammonium ions do not improve cell viability through increased Gln synthesis

**Fig. 4:**
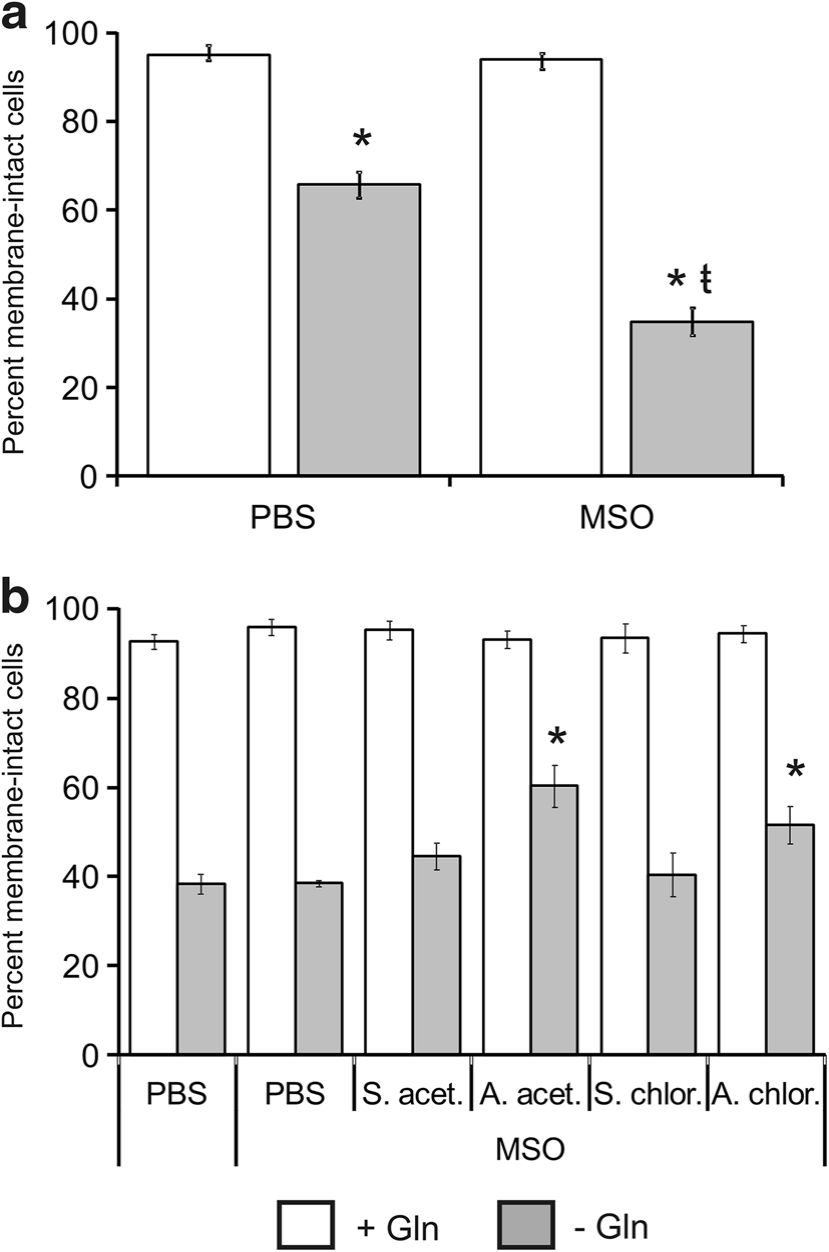
Effect of ammonium salts on the viability of Gln-starved Sp2/0-Ag14 cells does not depend on glutamine synthesis. **a** Ramos B cells were deprived of l-glutamine for 24 h in the presence or absence of methionine sulfoximine (MSO). Cell viability was then determined using the trypan blue assay. *p < 0.05 vs corresponding control cultured in the presence of Gln ŧ < 0.05 vs corresponding PBS control. **b** Sp2/0-Ag14 cells were deprived of glutamine for 3 h in the presence of MSO and of 5 mM ammonium salt/sodium salt or an equivalent volume of PBS. Gln (4 mM) was then added and culture was resumed for 24 h. Cell viability was determined by trypan blue exclusion. Gln-deprived, PBS-supplemented cultures in which MSO was omitted were used as controls. For both panels, cultures in which Gln was added at the start of the experiment were included as controls. *p < 0.05 vs corresponding sodium salt-supplemented control

Through the sequential action of glutamate dehydrogenase and Gln synthetase, cells convert αKG into Gln. In these two reactions, ammonium ions act as the source of amine groups [35]. To address the contribution of Gln synthesis to the increase in cell viability afforded by ammonium ions upon Gln starvation, we used the Gln synthetase inhibitor methionine sulfoximine (MSO) [36]. At the concentration used in this study (10 mM), MSO significantly sensitized the B lymphoma cell line Ramos to Gln starvation-induced cell death (Fig. 4a). When tested on Gln-deprived Sp2/0 cells, MSO did not result in a greater loss in cell viability over the PBS control (Fig. 4b). This was expected considering the low level of Gln synthetase activity in mouse hybridomas [37]. Importantly, the beneficial effect of ammonium salts on the viability of Gln-starved Sp2/0 cells was not blocked by MSO (Fig. 4b). Thus, the improvement in Sp2/0 cell survival afforded by ammonium ions does not require the synthesis of Gln.

### Non-additive effect of ammonium ions and αKG on the viability of Gln-starved Sp2/0 cells

**Fig. 5:**
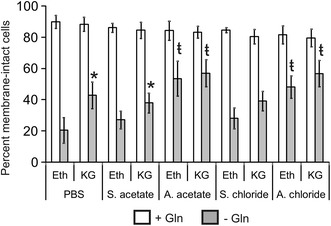
Non-additive effect of ammonium salts and α-ketoglutarate on the viability of Gln-starved Sp2/0-Ag14 cells. Cells were deprived of l-glutamine for 3 h in the presence of 2 mM dimethyl α-ketoglutarate (KG) and 5 mM ammonium salt/sodium salt or an equivalent volume of PBS. Cultures in which ethanol (Eth) was added in place of KG were included as controls. Gln (4 mM) was then added and culture was resumed for 24 h. Cell viability was determined by trypan blue exclusion. Cultures in which Gln was added at the start of the experiment were included as controls. *p < 0.05 vs corresponding ethanol-supplemented control. ŧ < 0.05 vs corresponding sodium salt-supplemented control

Considering recent reports showing that αKG improves the viability of Gln-starved cells [17], we tested the possibility that this metabolite could potentiate the effect of ammonium ions on the viability of Gln-starved Sp2/0 cells. When added to cultures of Sp2/0 cells deprived of Gln and supplemented with PBS, diMe α-KG (a cell-permeable precursor of αKG [38]) caused a limited but significant increase in the number of viable cells over the ethanol-treated control (43 vs 20 % viable cells) (Fig. 5). However, when used in combination with an ammonium salt, diMe α-KG did not result in a greater protection against Gln starvation-induced cell death compared to cells treated with AA or AC alone. Therefore, the two-end products of glutaminolysis, αKG and ammonium ions, contribute to the promotion of survival in Gln-deprived Sp2/0 cells in a non-additive fashion.

### Ammonium ions do not prevent core apoptotic events

Having established that ammonium ions improve the survival of Gln-deprived Sp2/0 cells, we next investigated their effect on the apoptotic machinery.

**Fig. 6:**
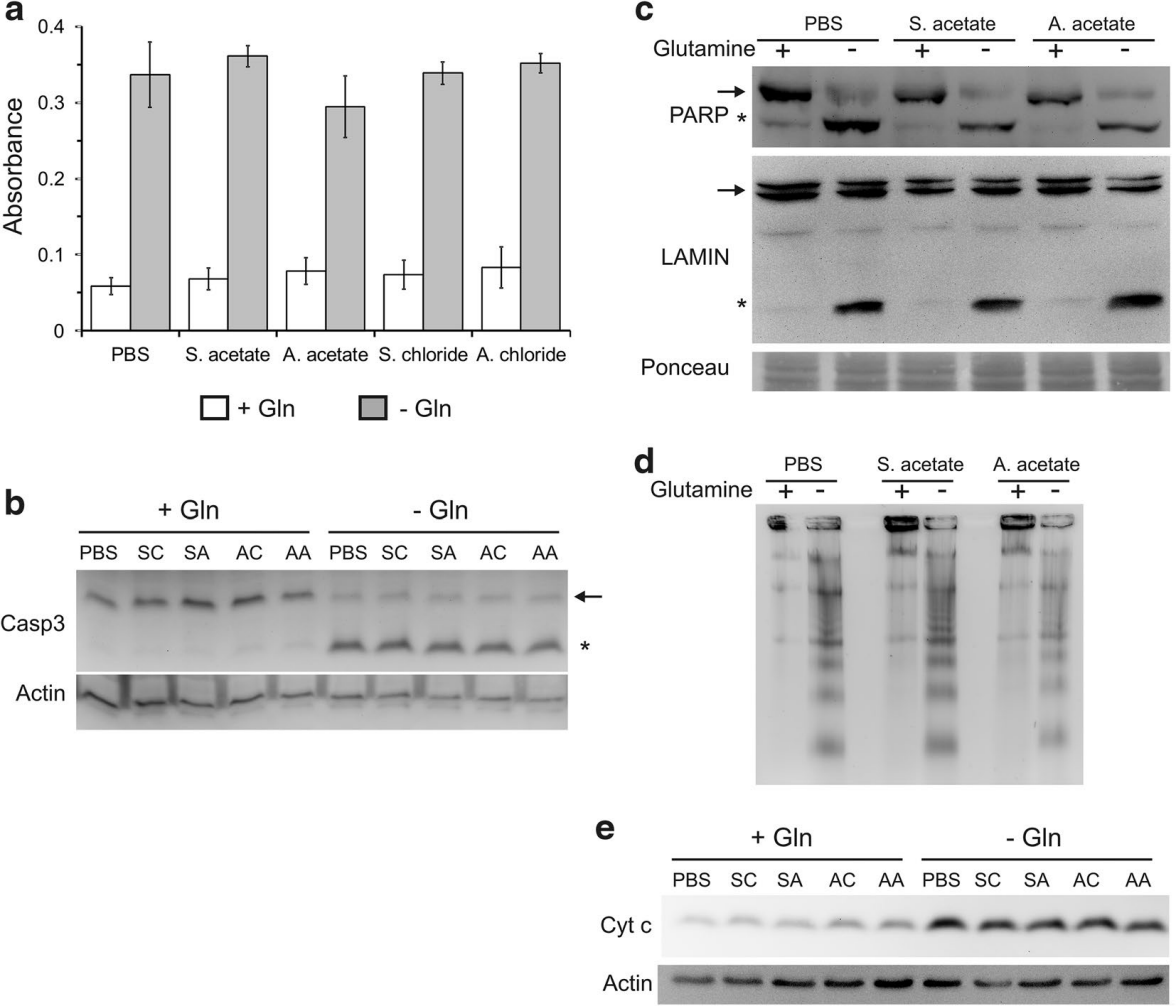
Ammonium salts do not interfere with the core apoptotic machinery. Sp2/0-Ag14 cells were deprived of l-glutamine (Gln) for 3 h in the presence of 5 mM ammonium salt/sodium salt or an equivalent volume of PBS. **a** Caspase-3 activity assay. **b** Caspase-3 processing. The *arrow* and the *star* indicate the unprocessed, inactive form and the cleaved, active form of caspase-3, respectively. **c** cleavage of PARP and Lamin A/C. The *arrow* and the *star* respectively indicate the full length and the caspase-3 cleaved forms of PARP and Lamin. Ponceau staining of the membrane was used as a loading control. **d** DNA fragmentation analysis. **e** cytosolic cytochrome c release. In *both panels* **b** and **e**, actin was used as a gel loading control. *SC* sodium chloride, *SA* sodium acetate, *AC* ammonium chloride. *AA* ammonium acetate

We first tested the ability of ammonium ions to modulate the activity of caspase-3, a protease that plays a key role in apoptosis by mediating the limited proteolysis of cellular proteins, leading to the morphological changes that characterize apoptotic cells [39, 40]. Gln starvation led to a significant increase in caspase-3 activity in the PBS-supplemented Sp2/0 cultures (Fig. 6a), which is in line with our previously published data [31, 34]. Similar results were obtained for the sodium salt controls. Unexpectedly, the addition of the ammonium salts to Gln-starved Sp2/0 cultures did not lead to a reduction in caspase-3 activation. These data were confirmed by the Western blot analysis of caspase-3 processing (Fig. 6b), the cleavage of the caspase substrates Lamin A/C and PARP (Fig. 6c) as well as genomic DNA fragmentation, which is dependent on caspase-3-mediated proteolysis of the inhibitor of caspase-activated DNAse protein (Fig. 6d) [39].

In Sp2/0 cells, Gln starvation rapidly triggers the activation of the intrinsic pathway, a phenomenon characterized by the permeabilization of the outer mitochondrial membrane and the release of cytochrome c from the mitochondrial intermembrane space into the cytoplasm [31, 34, 40]. Supplementation of Gln-deprived Sp2/0 cultures with AA or AC did not lead to a reduction in the cytosolic release of cytochrome c (Fig. 6e), indicating that the ammonium ions did not interfere with the onset of the intrinsic apoptotic pathway. Thus, the beneficial effect of ammonium salts on the viability of Gln-deprived Sp2/0 cells does not involve the inhibition of core apoptotic processes.

### Ammonium ions decrease apoptotic nuclear fragmentation and condensation

We have previously shown that inhibition of the p38 stress kinase in Gln-starved Sp2/0 cells results in a decrease in nuclear fragmentation and condensation, two late apoptotic events, without impeding cytochrome c release and caspase-3 activation [34]. Considering that neither AA nor AC inhibited the activation of the intrinsic apoptotic pathway (Fig. 6), we tested whether ammonium ions modulate the changes in nuclear morphology generally associated with apoptosis.

**Fig. 7:**
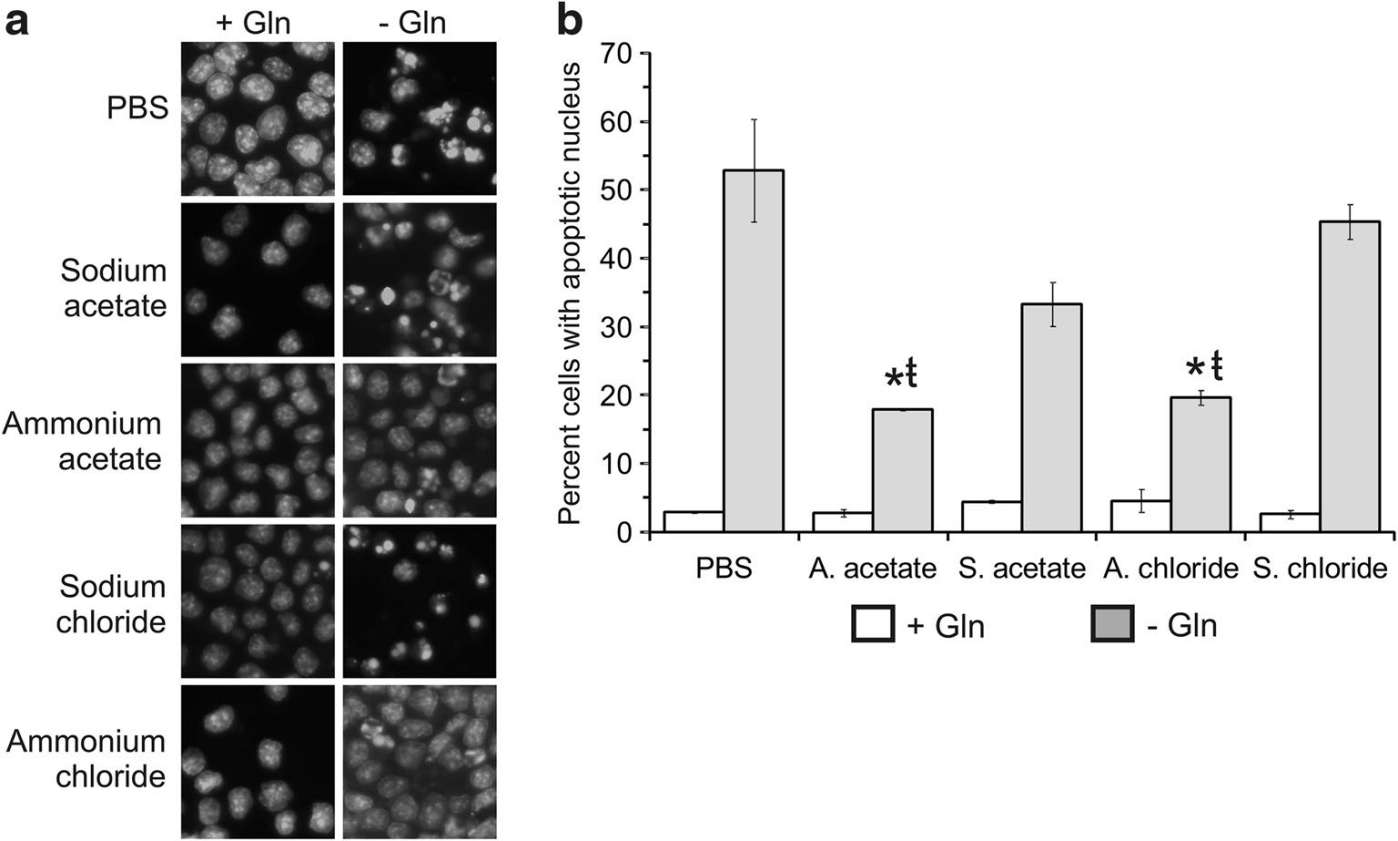
Ammonium salts interfere with apoptotic nuclear condensation and fragmentation. Sp2/0-Ag14 cells were deprived of l-glutamine (Gln) for 3 h in the presence of 5 mM ammonium salt/sodium salt or an equivalent volume of PBS. Hoechst 33342 was added to the cultures 30 min before the end of the experiment. The cells were then processed for fluorescence microscopy. **a** fluorescence microphotographs (magnification: ×400). **b** enumeration of cells showing apoptotic nuclear condensation/fragmentation. At least 200 nuclei were enumerated for each group. *p < 0.05 vs corresponding PBS-supplemented control. ŧ < 0.05 vs corresponding sodium salt-supplemented control

When Sp2/0 cells were deprived of Gln, supplementation with PBS or the sodium salts led to the expected changes in nuclear morphology typical of apoptosis: chromatin condensation was evident, as well as nuclear fragmentation (Fig. 7a). However, in the presence of AA or AC, a greater proportion of the Gln-starved Sp2/0 cells exhibited normal nuclear morphology. Indeed, the enumeration of apoptotic nuclei revealed that AA and AC caused a significant reduction in the proportion of Gln-deprived cells with apoptotic nuclear morphology (Fig. 7b). Therefore, ammonium ions protect Sp2/0 cells against Gln starvation-induced cell death through pathways that interfere with apoptotic nuclear condensation and fragmentation.

## Discussion

In this paper, we report on the effect of ammonium ions on the viability of Gln-deprived Sp2/0 cells. Using several assays, we show that the supplementation of Gln-starved Sp2/0 cultures with ammonium salts significantly increased cell viability, an effect that was not observed with the corresponding sodium salts. Moreover, we show that while ammonium ions did not impede the activation of the core apoptotic machinery, they did interfere with nuclear condensation and fragmentation.

While several studies have shown that ammonium ions trigger cell death [27, 28, 41–44], others have also revealed that they can play a role in the promotion of cell survival. In rat cerebellar neuron cultures exposed to glutamate, a chronic treatment with 100 µM ammonium salt caused a significant increase in cell viability [30, 45]. Interestingly, and unlike our observations with Sp2/0 cells, supplementing glutamate-treated cerebellar neurons with ammonium salts led to the inhibition of the intrinsic apoptotic pathway [30]. In a separate study, Eng et al. showed that ammonium ions protected iBMK cells against TNFα-induced cytotoxicity [29]. Moreover, and in contrast to the work of Llansola et al. [30] and the data presented in this report, ammonium ions improved the viability of TNFα-treated iBMK cells only when αKG was also supplied [29]. Therefore, although a limited number of studies have reported on a pro-survival function of ammonium ions, the evidence indicate that this effect is observed with a variety of cell lines subjected to different death triggers, and that different molecular mechanisms are likely at play.

The molecular pathways responsible for the modulation of the survival of Gln-deprived Sp2/0 cells by ammonium ions remain to be characterized. Our data indicate that, at least in the context of Gln-deprived Sp2/0 cells, ammonium ions impede nuclear fragmentation and condensation without interfering with caspase activation. The modulation of nuclear size by ammonium ions has been described in astrocytes [46], and ammonium ions have been shown to increase the activity of phospholipase A2 [47], an enzyme which was linked to nuclear shrinkage occurring during caspase-independent cell death [48]. Whether phospholipase A2 plays such a role in Gln-deprived Sp2/0 cells remains to be determined.

Ammonium ions interfere with a number of key cellular processes that can lead to perturbations in the cell death machinery. For instance, ammonium ions protect against glutamate-induced neuronal cell death by reducing the activation of the nitric oxide/cyclic GMP pathway and the increase in intracellular Ca^+2^ triggered by glutamate [30, 45]. The inhibition of TNFα-induced iBMK cell death depends on the ability of ammonium ions to stimulate autophagy [29]. The intracellular pool of UDP-N-acetylglucosamine and the N-acetylglucosamylation of several intracellular proteins have both been shown to be increased by ammonium chloride [49, 50]. This post-translational modification has been associated with the inhibition of cell death [51]. It is interesting to note that methylamine, which was ineffective in promoting the survival of Gln-deprived Sp2/0 cells (Fig. 2c), does not trigger the N-acetylglucosamylation of proteins [49]. Finally, because hydrated ammonium ions have an ionic radius similar to potassium ions, they interfere with K^+^ transport processes [52]. In fact, the major transporter of ammonium ions in hybridoma cells is the Na^+^/K^+^2Cl^−^ cotransporter [53]. The transport of ammonium ions into the cell is expected to hinder the efflux of K^+^ ions, a phenomenon central to the apoptotic process [54, 55].

Our data show that ammonium ions are not sufficient to completely inhibit the death program in Gln-starved Sp2/0 cells (Figs. 2, 3), indicating that other factors are required to ensure cell survival. One likely possibility is that glutamine metabolism leads to the formation of additional metabolites with pro-survival properties. This is supported by a number of observations. The addition of ammonia to hybridoma cultures triggers an increase in the activity of glutamate dehydrogenase, leading to the synthesis of glutamate from ammonia and αKG [56]. In addition to limiting the intracellular accumulation of ammonia, this was accompanied by the formation of aspartate, alanine and αKG, all through transamination reactions involving glutamate [56]. This is particularly interesting considering that substrates of the Krebs cycle (including αKG) as well as asparagine (which can be produced form aspartate and glutamine via the enzyme asparagine synthetase) have been shown to improve the survival of Gln-starved cells [17–19]. Moreover, αKG derived from glutaminolysis activates signaling through mTORC1, a signaling pathway known to play an important role in linking cell metabolism to autophagy and cell survival [14, 16]. Our observation that, when added to Gln-starved Sp2/0 cells, diMe α-KG caused a significant increase in cell viability (Fig. 5), certainly supports the idea that glutamine-mediated cell survival involves other metabolites in addition to ammonium ions. That the combination of diMe α-KG and ammonium salts did not have an additive effect on Sp2/0 cell survival (Fig. 5) may be due to the fact that our experiments were limited to using 2 mM diMe α-KG, as higher doses of this compound proved to be toxic to Sp2/0 cells.

## Conclusion

The metabolic reprogramming that characterizes rapidly proliferating cells has triggered a renewed interest in the manipulation of metabolic pathways for therapeutic purposes [57, 58]. In particular, the targeting of signaling pathways linking Gln to the cell survival machinery promises to yield cancer-specific therapies [6, 7, 58]. In that context, our demonstration that ammonium ions partially inhibit the Sp2/0 cell death program triggered by Gln starvation suggest that this major product of glutaminolysis should be considered as a potential target of anti-cancer strategies.

## Abbreviations

αKG: α-ketoglutarate
AA: ammonium acetate
AC: ammonium chloride
AnnV: annexin V-FITC
diMe α-KG: dimethyl α-ketoglutarate
Gln: l-glutamine
IMDM: Iscove’s-modified Dulbecco’s medium
MSO: methionine sulfoximine
PI: propidium iodide
SA: sodium acetate
SC: sodium chloride
Sp2/0: Sp2/0-Ag14
